# Crucial Cell Signaling Compounds Crosstalk and Integrative Multi-Omics Techniques for Salinity Stress Tolerance in Plants

**DOI:** 10.3389/fpls.2021.670369

**Published:** 2021-08-13

**Authors:** Rajesh K. Singhal, Debanjana Saha, Milan Skalicky, Udit N. Mishra, Jyoti Chauhan, Laxmi P. Behera, Devidutta Lenka, Subhash Chand, Vivek Kumar, Prajjal Dey, Indu, Saurabh Pandey, Pavla Vachova, Aayushi Gupta, Marian Brestic, Ayman El Sabagh

**Affiliations:** ^1^ICAR-Indian Grassland and Fodder Research Institute, Jhansi, India; ^2^Department of Biotechnology, Centurion University of Technology and Management, Bhubaneswar, India; ^3^Department of Botany and Plant Physiology, Faculty of Agrobiology, Food, and Natural Resources, Czech University of Life Sciences Prague, Prague, Czechia; ^4^Faculty of Agriculture, Sri Sri University, Cuttack, India; ^5^Narayan Institute of Agricultural Sciences, Gopal Narayan Singh University, Jamuhar, India; ^6^Department of Agriculture Biotechnology, Orissa University of Agriculture and Technology, Bhubaneswar, India; ^7^Department of Plant Breeding and Genetics, Orissa University of Agriculture and Technology, Bhubaneswar, India; ^8^Institute of Agriculture Sciences, Banaras Hindu University, Varanasi, India; ^9^Faculty of Agriculture, Sri Sri University, Cuttack, India; ^10^Department of Agriculture, Guru Nanak Dev University, Amritsar, India; ^11^Department of Plant Physiology, Slovak University of Agriculture in Nitra, Nitra, Slovakia; ^12^Department of Agronomy, Faculty of Agriculture, University of Kafrelsheikh, Kafr El Sheikh, Egypt; ^13^Department of Field Crops, Faculty of Agriculture, Siirt University, Siirt, Turkey

**Keywords:** antioxidant defense, crosstalk, homeostasis, omics approaches, signaling network, plant growth regulators, salinity stress tolerance

## Abstract

In the era of rapid climate change, abiotic stresses are the primary cause for yield gap in major agricultural crops. Among them, salinity is considered a calamitous stress due to its global distribution and consequences. Salinity affects plant processes and growth by imposing osmotic stress and destroys ionic and redox signaling. It also affects phytohormone homeostasis, which leads to oxidative stress and eventually imbalances metabolic activity. In this situation, signaling compound crosstalk such as gasotransmitters [nitric oxide (NO), hydrogen sulfide (H_2_S), hydrogen peroxide (H_2_O_2_), calcium (Ca), reactive oxygen species (ROS)] and plant growth regulators (auxin, ethylene, abscisic acid, and salicylic acid) have a decisive role in regulating plant stress signaling and administer unfavorable circumstances including salinity stress. Moreover, recent significant progress in omics techniques (transcriptomics, genomics, proteomics, and metabolomics) have helped to reinforce the deep understanding of molecular insight in multiple stress tolerance. Currently, there is very little information on gasotransmitters and plant growth regulator crosstalk and inadequacy of information regarding the integration of multi-omics technology during salinity stress. Therefore, there is an urgent need to understand the crucial cell signaling crosstalk mechanisms and integrative multi-omics techniques to provide a more direct approach for salinity stress tolerance. To address the above-mentioned words, this review covers the common mechanisms of signaling compounds and role of different signaling crosstalk under salinity stress tolerance. Thereafter, we mention the integration of different omics technology and compile recent information with respect to salinity stress tolerance.

## Introduction

**FIGURE 1:**
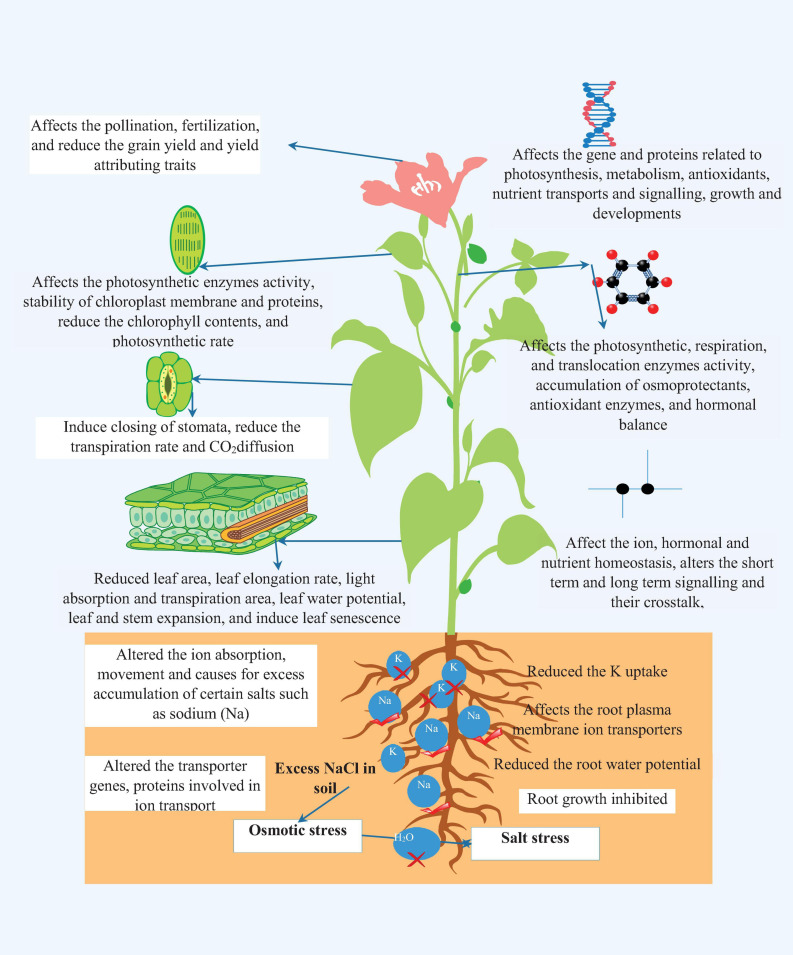
The effect of salinity stress on root growth, ionic homeostasis, physiological, biochemical, and molecular processes.

Soil is an indispensable component of the environment and a fundamental prerequisite for nourishing optimistic plant growth and development. Along with its supporting role, soil provides essential nutrient and mineral elements for the vigorous and productive growth of plants. Despite this, the extensive use of chemical fertilizers, excess irrigation, farm mechanization, and other anthropogenic activities act as stress factors for soil natural properties and lead to soil salinity (Pessarakli and Szabolcs, 1999). Soil salinity is one of the global indispensable stress factors, affecting plant growth drastically in both irrigated and rain-fed areas (Hussain et al., 2019; Sabagh et al., 2019; Liu L. et al., 2020). According to the Land and Plant Nutrition Management Service report, approximately 6% (>45 Mha) of the world’s cultivated areas and about one-third of irrigated land on earth is affected by the salinity stress (Carillo et al., 2011; Deinlein et al., 2014; Parihar et al., 2015). Soil salinity situations emerge when glut salts leach and accrue in soil and at the same time, there is no room to flush out the accumulated salts to a well-managed drainage system (Qadir et al., 2008). At the same time, salt concentration swiftly reaches levels that are injurious to salt-sensitive species and beyond this, salt concentration levels can also affect salt-tolerant species. The initial stage of salinity stress is osmotic stress and perused by ion toxicity, which mainly targets uptake and transport of essential ions in plant roots (Serrano and Rodriguez-Navarro, 2001). Salinity stress induces considerable changes in physiological, biochemical, and molecular processes, depending on the extent and severity of the stress, types of genotypes, and crop stages which ultimately leads to huge yield penalty in important agricultural crops (Zeng et al., 2001; Thitisaksakul et al., 2015; Negrão et al., 2017). Osmotic stress culminates in loss of water absorption capacity of the root system, water potential of leaves, membrane damage, nutrients inequity, reduced photosynthetic and metabolic processes, and abatement of the antioxidant defense of plants (Amirjani, 2010; Yan et al., 2013; Parihar et al., 2015). Severe toxicity leads to alterations in crucial plant processes and destroys root functions via modifying redox potential, ion homeostasis, hormonal balance, transpiration, and generates a high amount of ROS [singlet oxygen, superoxide, hydroxyl radical, and hydrogen peroxide (H_2_O_2_)], which damages the cellular membrane integrity and macromolecular structure (carbohydrate, proteins, lipids, and DNA) (Läuchli and Grattan, 2007; Farkhondeh et al., 2012). The consequences of salinity on root and plant functions, fundamental processes, and at molecular levels are illustrated in Figure 1.

Hence, salt stress adaptivity or tolerance responses are very crucial to develop stress-tolerant varieties under unfavorable situations. As a consequence, plants activate some exclusive physiological, biochemical, and molecular mechanisms in order to survive under stress conditions, which involve the activation of antioxidant enzymes, compartmentalization, uptake and transport of ions, accumulation of osmoprotectants and compatible solutes, ion homeostasis, and secondary metabolites (Gupta and Huang, 2014; Singhal et al., 2017). Nevertheless, the natural capacity of plants enable them to achieve the desired food potential under these circumstances. Therefore, researchers are continuously working on finding strategies and mechanisms to boost their final potential. In this regard, identifying differentially expressed genes and gene products and transgenic approaches that are associated with stress tolerance are promising approaches to develop smart crops (Yang and Guo, 2018). Even if, due to the complex nature and effects of abiotic stresses on plant processes, these strategies are also completely efficient to achieve the goal of food security under climate change. Therefore, recently scientists have been working on finding and elucidating sophisticated signaling and molecular approaches to develop multiple stress-tolerant crops.

At this point, actuating the signal-transduction cascade for versatile climate plant responses includes various adjustments that are produced in an exceptionally well-coordinated way for exhibiting great opportunities to provide stress tolerance. The promoter-reporter approach has also been exceptionally helpful for identifying genes involved in osmotic stress (Ishitani et al., 1997), and has significantly improved salt-stress monitoring in higher plants. Ionic homeostasis under salt stress is mediated by the SOS (salt overlay sensitive) pathway in a Ca^+2^-dependent manner, which transduces the salt stress signal in a regulated pattern with the SOS3-SOS2 protein kinase complex at the cell membrane that adds an Na^+^ ion into the cell and balances ion homeostasis (Ji et al., 2013; Gupta and Huang, 2014). Interestingly, several other signaling compounds such as nitric oxide (NO), hydrogen sulfide (H_2_S), H_2_O_2_, Ca, ROS, and plant growth regulators salicylic acid (SA), jasmonic acid (JA), ethylene (ET), and abscisic acid (ABA) have crucial roles during cell signaling and crosstalk as they provide tolerance to multiple stresses (Chauhan et al., 2017; Noctor et al., 2018; Pei et al., 2018). Moreover, germplasm resources and integrated “omics-assisted” approaches such as phenomics, ionomics, transcriptomics, proteomics, genomics, miRNAomics, lipidomics, and metabolomics are prominently used for developing salt tolerance in crop species (Ho et al., 2020). Correspondingly, epigenetics and next generation phenotyping also provide efficient platforms in context to the production of salt stress-tolerant species (Jha et al., 2019). All integrated omics-assisted approaches have contributed extraordinarily to understanding the outcomes of salinity stress and the alterations which plants adopt for survival and mitigation under unfavorable conditions (Mehta et al., 2019; Pan et al., 2020; Rasel et al., 2020). It is important to keep in mind that, in the present perspective, we point out the recent advances in the crosstalk of important signaling compounds and their role under salinity stress tolerance. Further, we address the recent advances in integrative multi-omics approaches, which are crucial to provide salinity tolerance and a future platform to develop promising salt-tolerant varieties for salt stress conditions.

## Plant Signaling Compounds and Crosstalk Mechanisms Under Stress Regulations

Plants under stressed conditions such as abiotic (heat, cold, salinity, heavy metal) and biotic stresses must be acknowledged and the innate immune system must be activated for survival and better fitness. The survival of plants under stressful conditions depends on compact signaling networks and their crosstalk (Tena et al., 2011; Smékalová et al., 2014). Signaling pathway activities are activated after the sensation of a signal produced by a specified receptor that triggers the urging of secondary signals and protein phosphorylation cascades like MAPK signaling. Secondary messengers such as ROS, Ca^2+^, NO, H_2_S, H_2_O_2_, phospholipids, and PGRs act as secondary signals during signaling cascades (DeFalco et al., 2010; Suzuki et al., 2012). These signals are involved in signaling pathways through multiple ways and administer fundamental processes such as cell division and growth, differentiation, and programmed cell death under normal as well as abiotic and biotic interactions (Müller et al., 2010; Tena et al., 2011; Sasabe and Machida, 2012).

Salt stress changes to membrane structures induce metabolic stress, form ROS, and prevent photosynthesis leading to nutrient deficiency (Hasegawa et al., 2000; Tuteja, 2007). The growth responses to salinity include two distinct stages (Munns, 1993). Hormonal signals from the root’s delay development, and then switch off the signal when the plant is mature. Growth reduction can be attributed to a salt-specific effect, which often takes a while (varies between weeks to years) to create. The second step is a result of decreased water availability and accumulation of salt in transpiring leaves, adding to thresholds that surpass the capacity of a cell to sequester salts into vacuoles (Munns, 1993, 2005; Läuchli and Grattan, 2007). Na^+^ reaches cells quite quickly just after the occurrence of salt stress. The increased sodium in this water might influence its salinity adaptation. Therefore, the crucial mechanism of mitogen activates protein kinase and Ca signaling under stress conditions are discussed in the next section.

### Mitogen-Activated Protein Kinase Signaling (MAPK)

The function of MAPK relies on post translational phosphorylation signaling, established by a serine/threonine kinase, i.e., mitogen-activated protein kinase kinase kinase (MAPKKK or MAP3K) that reversibly phosphorylates MAPKK (a dual-specificity kinase), then that phosphorylates MAPKs (Keshet and Seger, 2010; Smékalová et al., 2014). MAPKs are involved in phosphorylation of transcription factors, cytoskeleton-associated protein, and protein kinase in plants (Nakagami et al., 2005). Recently, various researchers established that root growth initiation was due to the activity of Ca^2+^ channels and production of auxin which boost the accumulation of NO. This NO is culpable for the modulation in Ca^2+^ channel movement and MAPK cascade enzyme activities. ROS molecules, which form as metabolic by-products under stressed conditions, can also induce the activation of MAPKs (Nakagami et al., 2005).

Plant hormones act as an impressive signaling molecule under both normal and stress conditions. MAPK cascades also respond to various hormone signaling like auxin, SA, JA, brassinosteroids (BRs), strigolactones, ABA, and ET. These signaling molecules accomplish a distinct signaling network, which crosstalk to each other and respond under normal and stress conditions (Devoto and Turner, 2003; Depuydt and Hardtke, 2011; Chini et al., 2016). Treatment with natural and synthetic auxin triggers the prompt actuation of MAPKs in the roots of Arabidopsis (Mockaitis and Howell, 2000). ABA signaling has a massive role in the plant growth process that deals with the turgor and stomatal activity of plant cells. MPK4, MPK9, MPK12, and MPK15 proteins exist in guard cells (Zhao et al., 2008), which exhibit the decisive role in ABA signaling and are possibly associated with the activation of the ABA-dependent anion channel (Jammes et al., 2009). Under stress conditions, ABA induces the production of H_2_O_2_ and the expression of catalase (CAT) isoform CAT1. This expression is mediated by Arabidopsis MAP2K, in response to H_2_O_2_ (Xing et al., 2008). H_2_S is considered an endogenous gaseous transmitter that exhibits a specific role in the germination of seed, root growth, stomatal activity, photosynthesis, and abscission of plant organs under normal as well as stressed conditions (Corpas and Palma, 2020). H_2_S interacts with other signal molecules such as ABA, ethylene, auxin, Ca^2+^, CO, and NO, and controls post transitional modification of proteins (Hancock and Whiteman, 2016; Xuan et al., 2020). ABA induced H_2_S accumulation via activating SnRK2.6 activities at Cys131 and Cys137 by *S*-sulfhydration of SnRK2.6 that enhances the interaction of SnRK2.6 with ABA responsive element-binding factor ABF2 (Chen S. et al., 2020). NO is involved in ABA and ethylene crosstalk (Domingos et al., 2015). Indeed, NO was produced during the initial phase of seed germination and promoted seedling growth by inducing ABA 8′-hydroxylase gene expression and ethylene production. Ethylene protects the Brassicaceae seed from the inhibitory effect of ABA by stimulating weakening and rupturing seed testa and endosperm (Arc et al., 2013). NO donors inhibit ethylene biosynthesis and prevent the dormancy of seeds and stimulate germination in apples (Gniazdowska et al., 2007). Breaking of apple seed dormancy by NO encourages ROS production, which stimulates ethylene accumulation due to an increase in ACS and ACO activity (Gniazdowska et al., 2010). EREBPs, which are described as transcriptional factors induced by NO, and ethylene stimulate EREBP-3 just before the rupturing of endosperm during tobacco seed germination, which is inhibited by ABA (Leubner-Metzger et al., 1998). H_2_S effectively alleviated ethylene-mediated fruit softening in Kiwi fruits and enhanced the ascorbic acid, starch, sugar protein, and titratable acidity (Li T. T. et al., 2017). Combined treatment of H_2_S-ET inhibited ET synthesis and its related genes such as ACS6, ACO1, ACO4, ERF1, and ETR4, thus suppressed ET induced petiole abscission in tomatoes (Liu D. et al., 2020). Treatment with a higher level of NaHS inhibited primary root growth, initiated by ROS and NO accumulation and activation of the MPK6 gene (Zhang et al., 2017), which denoted that ROS-MPK6-NO cascading intermediates have repressive impacts of high concentration of H_2_S on root activity (Zhang et al., 2017). H_2_S strengthens the plant capacity to heat and aluminum (Al) tolerance by reducing oxidative damage after interaction with NO (Sun et al., 2016). Both H_2_S and NO interactions improved the survival rate of plants under heat stress conditions, due to a decrease in malondialdehyde (MDA) accumulation and enhanced antioxidant capacity in maize and strawberry (Uchida et al., 2002; Christou et al., 2014; Li J. et al., 2014; Li Z. G. et al., 2014).

### Calcium (Ca^2+^) Signaling

Under salinity stress, plants depict two forms at the same time including osmotic and ionic stresses. “Cell apoptosis versus adaptation” is dependent on the timing of two cellular responses: the first prompted by Ca, and the second prompted by oxidative outburst in the apoplast. A delay in the formation and dissipation of a salinity-triggered Ca-dependent signal coupled with ROS activates JA signaling, leading to the death of the cells. In contrast to the same molecular signal, calcium will, when properly timed, activate various adaptive processes including sequestration and extrusion of sodium, and also through ABA signaling. With respect to the perception of external inputs, calcium transients, from a number of extracellular compartments, become cytosolic through transient Ca^2+^-dependent Ca^2+^ channels. The “Ca^2+^-signature/Ca^2+^-spiking” concept became common when it was defined by Webb et al. (1996). The pattern of calcium signaling is determined by the type and amplitude of the stimuli. The calcium level in the body has an enormous impact on the success level of life forms. Calcium-binding proteins, functioning as calcium receptors, relay the information to be conveyed from Ca signals. An unprecedented rate of sensitivity is accomplished by a group of calcium binding modules, that include ‘calmodulin’ (CaM), ‘calmodulin-like protein (CML) family,’ ‘Ca^2+^-dependent protein kinases’ (CDPK), Ca^2+^-binding proteins serving as “Ca^2+^ sensors,” ‘calcineurin B-like proteins (CBLs),’ “Ca^2+^-decoders,” and ‘CBL-interacting protein kinases (CIPKs)’ which all together transmit the information embedded within calcium signatures. CaM is conserved regardless of species, while CML, CDPK, and CBL are unique to plants and some prokaryotes (Day et al., 2002; Harper and Harmon, 2005; Batistič and Kudla, 2009). Single-cell systems, including pollen germination, provide an excellent model to unveil the coding mechanism and determinants of “Ca^2+^-signature.” Induction of calcium transients mainly occurs at the organ level *via* a single spike. The induction of “Ca^2+^-signature” is in accordance with (i) Ca^2+^ in various plasma membrane (PM) and endomembrane (EM) flux channels, (ii) cytosolic Ca^2+^ rallying (in and out) induced by Ca^2+^ influx and efflux transporters, respectively (McAinsh and Pittman, 2009; Kudla et al., 2010). Therefore, plant signaling is very complex in nature and numerous signaling compounds regulate the plant processes under normal and stress conditions.

## Signaling Compounds Crosstalk During Salt Stress Tolerance

**FIGURE 2:**
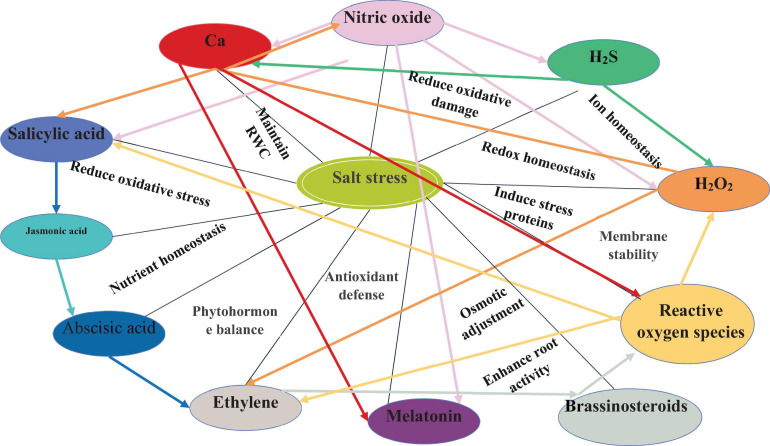
The crosstalk of signaling compounds and function regulated under salinity stress.

Salt tolerance is very complex in nature and affects various processes in plants. In this regard, signaling compounds such as NO, H_2_S, H_2_O_2_, ROS, and plant growth regulators crosstalk with each other and coordinate numerous plant functions and processes, which are associated with salinity tolerance. The crosstalk of various signaling compounds for salinity tolerance are discussed, followed, and represented in Table 1 and Figure 2.

**TABLE 1:** The crosstalk of crucial signaling compounds under salinity stress and their salt tolerance mechanism in different crops.

Crosstalk	Crop	Salt tolerance mechanism	References
GST-NO	*Glycine max* L.	NO induces the GST1 and GST4 isoenzymes and transcript levels in ABA-dependent and independent pathways	Dinler et al. (2014)
NO-CaCl_2_	*Brassica juncea* L. cv. Varuna	Enhances the antioxidant enzymes activities (SOD, CAT, APX, GR, and POX), osmoprotectant (proline and glycinebetaine), and nutrient homeostasis (increase leaf K^+^, Ca, and decrease Na^+^). Combined application reduces oxidative stress by decreasing H_2_O_2_ content and lipid peroxidation	Khan et al. (2012)
H_2_O_2_-NO	*Oryza sativa* L. cv. Nipponbare	Improves antioxidant enzymes activity, and induces the expression of sucrose phosphate synthase (SPS), Δ′-pyrroline-5-carboxylate synthase, and HSP26	Uchida et al. (2002)
H_2_O_2_-NO	*P. euphratica* and *P. popularis*	Improves antioxidant defense by activating antioxidant enzymes, reduces oxidative stress, and maintains redox and nutrient homeostasis	Sun et al. (2010)
H_2_S-NO	*Medicago sativa* L., Victoria	Induction of APX1, APX2, Mn-SOD, Fe-SOD, Cu/Zn-SOD isoforms transcripts level, and re-establishment of ion homeostasis	Wang et al. (2012)
H_2_S-H_2_O_2_	*Arabidopsis thaliana*	Promotes the expression and phosphorylation of PM H^+^-ATPase and Na^+^-H^+^ antiporter protein, and regulates the activity of G6PDH and PM NADPH oxidase in roots	Khan et al. (2020)
H_2_S-H_2_O_2_	*Vicia faba*	Increased L/D cysteine desulfhydrase activity and induction of stomata closing	Ma et al. (2019)
H_2_O_2_-NO	*Citrus aurantium* L.	Prevents the modification in accumulation levels of crucial enzymes in the Calvin-Benson cycle, switches ‘on’ the antioxidant immunity system, prevents protein carbonylation, protects plant metabolism by regulating the enzymes in mitochondria, and protein reprogramming by prevention of NaCl responsive proteins	Tanou et al. (2009)
H_2_O_2_-NO-Ca	*Bruguiera gymnorrhiza* and *Kandelia candel*	Maintains ion flux and K^+^/Na^+^ ion homeostasis	Lu et al. (2013b)
NO-H_2_S	*Capsicum annuum* L	Improves total, shoot, and root biomass, decreases oxidative stress by reducing H_2_O_2_ production, prevents electrolyte leakage and MDA content, promotes CAT and SOD antioxidant activity, and maintains ion homeostasis	Kaya et al. (2020)
Ca-ROS	*Arabidopsis thaliana* and Halophytes	Promotes cytosolic ion balance and downstream signaling in activation of antioxidant enzymes	Kurusu et al. (2015)
H_2_O_2_-NO-Ca	*Chenopodium quinoa*	Induces amylase activity, seed reserve hydrolysis, accumulation of water-soluble sugar, and enhances protein and amino acid contents in seedlings	Hajihashemi et al. (2020)
Ca-H_2_S	*Vigna radiate*	Promotes ion homeostasis, improves transport of nutrients, reduces oxidative damage, and induces antioxidants defense and proline metabolism	Khan et al. (2020)

*Ψ HSP, heat shock protein; G6PDH, glucose-6-phosphate dehydrogenase; ROS, reactive oxygen species; CAT, catalase; SOD, sodium dismutase; MDA, malondialdehyde; APX, ascorbate peroxidase; POX, peroxidase.*

### NO Crosstalk

Nitric oxide is the primary gasotransmitter, administering numerous physiological and signaling functions, and also promotes salinity tolerance in plants (Xie et al., 2008). In recent years, it was confirmed that NO crosstalk with other signaling compounds and phytohormone signaling pathways helps in alleviating salinity stress (Tanou et al., 2009; Poór and Tari, 2011; Zhao et al., 2018). Auxin (AUX), ET, and ABA are imperative plant hormones that move from salt-treated roots to leaves that induce synthesis of NO or are transported throughout the plant (Molassiotis et al., 2010). Further, an increase in antioxidant activity and a decrease in thiobarbituric acid, which is reactive material content, is associated with NO-induced alleviation of oxidative damage in saline areas (Xu J. et al., 2011). In cotton, supplying exogenously NO (using SNP sodium nitroprusside for the NO donor) reduces the salt-induced senescence in leaves through downregulating the manifestation of ABA biosynthesis genes such as *NCED 9* (9-*cis*-epoxycarotenoid- dioxygenase) and *NCED 2* (Kong et al., 2016). In Arabidopsis, a callus treated with 100 mM of NaCl stimulated NO accumulation that added to ET emission, and resulted in induction of H^+^-ATPase gene expression in the plasma membrane (PM) (Wang et al., 2009). However, an antagonistic relationship between NO and ET in a suspension culture of tomato cells treated with 100 and 200 mM of NaCl was reported and suggested that an increase in ET synthesis encourages ROS production that is associated with high dead cell ratio, whereas production of NO curtails the dead cell ratio (Poór and Tari, 2011). In the cell suspension culture and segments of apical root, both lack NO and ET-generated (Na^+^/K^+^) ionic imbalance, respectively, that leads to an increase in susceptibility toward salinity stress (Poór et al., 2011). Application of SA and SNP, in combination, reduces NaCl-induced toxicity by supplementing the accumulation of proline and stimulation of GPX (glutathione peroxidase), CAT (catalase), and APX (ascorbate peroxidase) in soybean seedlings (Simaei et al., 2011). SA interaction along with signaling flow of NO alters the photosynthetic capacity along with diminished accumulation of H_2_O_2_, which enhances the influx of H^+^-ATPase into PM. The collaborative effect of both SA and NO promotes the absorption of Ca^2+^/Mg^2+^ with decreased Na^+^ uptake in saline conditions (Dong et al., 2015).

Among metabolites, sulfur is the main constituent present in reduced glutathione (GSH), methionine, coenzyme A, cysteine (Cys), iron–sulfur (Fe–S), thioredoxin, and sulfo-lipid systems associated with regulating the physiological process in salt stress environments (Khan et al., 2013). Further, NO enhances S-assimilation which is linked with ET synthesis through cysteine production. Sulfur and NO interact to regulate ABA and ET level in the guard cell and regulate photosynthetic and stomatal activities under salt conditions (Fatma et al., 2016). NO acts as a crucial regulatory signal, which activates various biochemical activities and their interaction with the sulfhydryl and nitro class during nitration enhances tolerance against salinity (Leterrier et al., 2011). NO along with other signaling compounds like H_2_S helps in building tolerance toward salinity stress in plants. It is observed that exogenous application of NO under saline conditions alters proline (Pro) metabolism and enhances the ratio of free proline accumulation that maintains the turgor potential and protects cucumber seedlings from salinity (Fan et al., 2012). In mustard, CaCl_2_ and/or SNP application alleviates salt stress by influencing antioxidant enzyme activities along with promoting glycinebetaine (gb) and proline (pro) accumulation, which is associated with a decrease in H_2_O_2_, TBARS (thiobarbituric acid reactive substances), and electrolyte leakage (Khan et al., 2012). In *Lactuca sativa*, application of NaCl triggers osmotic, oxidative, and ionic stress that arise into hormonal imbalances and reduced growth of the plant. Exogenous NO application results in reduction of Na^+^ accumulation, balancing the concentration of mineral nutrient, which is associated with balanced photosynthetic rate along with the established growth (Campos et al., 2019). Through NO signaling, phytohormone balance leads to osmotic regulation and also activates the antioxidant system with subsequent increase in tolerance level against salinity. The corm of *Crocus sativus* treated with NO shows more growth under salt-stress and promotes biosynthesis of the secondary metabolites, deposition of compatible solutes, and accelerates antioxidative enzyme activity, whereas treatment with SA did not boost plant growth during salinity (Babaei et al., 2021). Under saline conditions, NO-releasing substances and melatonin application counteracted inhibition of NaCl-treated seedling growth in addition to redox and ion homeostasis which is proved by retardation of ROS overproduction, Na+/K+ ratio, and reduction in the synthesis of TBARS. Consequently, increased level of NO augments addition of melatonin in seedling roots under salt stress (Zhao et al., 2018). Therefore, NO acts as an important signaling network with different signaling factors in plant systems under salinity stress.

### H_2_S Crosstalk

Hydrogen sulfide is a signaling molecule, which plays a major role in adventitious rooting, postharvest senescence, and seed germination (Deng et al., 2020), and provides a protective response toward multiple abiotic and biotic stresses (Corpas, 2019). Salt tolerance is enhanced through H_2_S by increased soluble protein content and chlorophyll under saline conditions but also inhibits ROS accumulation (Mostofa et al., 2015a). H_2_S donors which are identified/synthesized include CaS_2_, morpholin-4-ium 4-methoxyphenyl (morpholino) phosphinodithioate (GYY4137), sodium hydrosulfide (NaHS), NOSH-aspirin, dialkyldithiophosphate (ZDDP), AP39, and diallyl trisulfide (DATS). NOSH-aspirin releases two gasotransmitters simultaneously, H_2_S and NO (Kodela et al., 2012).

In plants, ROS levels are regulated by two ways, by scavenging excess ROS through antioxidant substances (like glutathione and ascorbate) and antioxidant enzymes linked with the AsA–GSH (ascorbate–glutathione) cycle. Accumulation of ROS is reduced by external application of H_2_S because H_2_S promotes antioxidant enzyme activities like SOD and CAT in Chinese cabbage (Zhang et al., 2015). From this we can assume that activity of antioxidant enzymes may be controlled by H_2_S through their protein expressions, thus decreasing accumulation of ROS due to Al toxicity. It can also maintain membrane integrity and ROS homeostasis by controlling the antioxidant mechanism (AsA-GSH cycle and enzymes), therefore enhancing the tolerance level in plants toward salinity stress. In recent studies, by maintaining Na^+^/K^+^ homeostasis, H_2_S helps in enhancing plant salt tolerance level. Under salinity conditions, the content of cellular Na^+^ mainly increased whereas K^+^ content reduced in rice, which shows a hike in Na^+^/K^+^ ratio in leaves and roots of rice. Therefore, exogenous H_2_S application maintains Na^+^/K^+^ homeostasis in saline conditions in rice (Mostofa et al., 2015b).

Along with NO as a molecule for signaling, H_2_S is also included in stress responses as they exist in a synergistic relationship (Hancock and Whiteman, 2016). The upstream and downstream relationship between H_2_S and NO focuses on two factors: NO involvement in the stress tolerance enhances H_2_S in plants and NO-induced stress tolerance where H_2_S acts as a downstream signal molecule. Nitrosothiol is a new compound produced when H_2_S reacts with NO and also results in a decrease of both the compound levels (Huang et al., 2020). In Arabidopsis roots, treatment with 100 mM of NaCl causes electrolyte discharge and also disturbs the Na^+^/K^+^ ratio but post treatment with NaHS promoted tolerance toward salt in roots (Li et al., 2016). Production of H_2_S in alfalfa plants boosted the tolerance level against salinity in the germination stage of seeds which may be induced through oxidative damage (Wang et al., 2012). H_2_S and NO modify the activity of many antioxidant enzymes like CAT, APX, and SOD to scavenge ROS which deceases their accumulation, thus helping in enhancing the tolerance level of plants toward higher salinity conditions (da-Silva et al., 2018).

Hydrogen sulfide interaction with various phytohormones such as ET, MT, ABA, and SA plays a fundamental role in response to abiotic stress (Huang et al., 2020). Gene expression related to ABA metabolism is increased by the exogenous H_2_S along with an upregulation of ABA receptor expression levels in roots of wheat during stress conditions, which indicates that the stress tolerance of wheat is promoted by H_2_S through the involvement of the ABA signaling pathway (Ma et al., 2016). When production of NO is restricted, ET is reduced which enhances the level of H_2_S. A study on *Vicia faba* L. showed that H_2_S biosynthesis inhibitors do not block the stomatal closure which is ET-induced with NO accumulation (Liu et al., 2016). Through the activation of endogenously synthesized H_2_S, SA enhances the tolerance level of plants toward several other abiotic stresses. Crosstalk of NO and H_2_S plays a crucial role in promoting the tolerance level against abiotic stresses. H_2_S not only regulates the uptake along with transport of metal ions and maintains Na^+^/K^+^ homeostasis during salinity but also shows an interaction with different signaling molecules such as Cys, phytohormones, and NO for enhancing the plant tolerance toward salinity stress.

### H_2_O_2_ Crosstalk

Hydrogen peroxide is synthesized in a form of ROS and as a result of oxidative stress that cause damage due to excess accumulation of ROS under different stress conditions, which results in the death of cells (Fotopoulos et al., 2006). H_2_O_2_ production is induced in plants following exposure to a different range of environmental stimuli. Further, it was found that H_2_O_2_ acts as a signal to determine different physiological, biochemical, and molecular responses within plants and cells and their crosstalk in between other signaling pathways (Neill et al., 2002). NO and H_2_O_2_ signaling pathways are coordinated and tightly linked to different plant responses toward the environmental stimuli (Molassiotis and Fotopoulos, 2011). Alteration in production of both NO and H_2_O_2_ is considered under these plant responses toward salt stress (Zhang et al., 2006). Further, it was reported that expression of the *AtNOA1* gene in Arabidopsis was suppressed by NaCl treatment which leads to a reduced NO level (Zhao M. G. et al., 2007). In contrast, expression of *OsNOA1*, the rice homolog of *AtNOA1*, was moderately enhanced due to salinity (Qiao et al., 2009). By pre-treating wheat seeds with H_2_O_2_, the salt tolerance level of the seedlings was improved (Wahid et al., 2007). Apart from the biosynthetic level, H_2_O_2_ and NO crosstalk are also included in protein activity and regulation of gene expression (Qiao et al., 2014). In Bermuda grass, NO and H_2_O_2_ regulate induction of CAT and SOD activity through ABA (Lu et al., 2009). From further studies, it has been observed that both ABA-dependent NO and H_2_O_2_ influenced the activity of antioxidant enzymes and transcription under salt tolerance (Zhang et al., 2009). Consequently, SA enhances endogenous H_2_O_2_ levels significantly through regulating activation of SOD. Therefore, SA and H_2_O_2_ work together in a self-amplifying process (Rao et al., 1997). The high concentration of H_2_O_2_ causes oxidative/nitrosative stress, while in low concentrations H_2_O_2_ acts as a signaling molecule to regulate stress responses.

### Ca Crosstalk

Calcium is an important signaling molecule, and secondary messenger crosstalk with several other signaling compounds help in the mitigation of salinity stress. Several studies have shown that flavonoids and phenylalanine ammonia lyase (PAL) activity are elevated after adding calcium chloride or an ionophore to the nutrient medium of a plant species (Paranhos, 2014). Ca^2+^-mediated modulation for the development of the specialized metabolites is collectively induced by JA(Lee-Parsons and Ertürk, 2005), ABA (Vighi et al., 2019), and SA (Guo H. et al., 2015). The effects of MT and Ca, and how they affect the development of phenolic compounds (PCs), were investigated in the plant *Dracaena kotschy*i under stress conditions of salt water and compared with control. Vafadar et al. (2020b) reported that external NaCl application (mimicking salinity stress) reduces dry biomass of shoots but elevates H_2_O_2_ content, electrolyte leakage (EL) level, and 2,2-diphenyl-1-picrylhydrazyl (DPPH) scavenging ability, and upregulates gene expression of PAL, RAS, and TAL enzymes. Vafadar et al. (2020a) found that pre-treatment of *D. kotschyi* with a Mel biosynthetic pathway inhibitor has no effect on Ca^2+^-mediated production of PCs in salt-affected plants. On the contrary, pre-treatment of *D. kotschyi* with a plasma membrane channel blocker, Ca^2+^ chelator, and calmodulin (CaM) antagonist resulted in impairment of Mel effects under salinity stress. This suggests that biosynthetic triggering of phenolics is attributed to Mel application only when influx of Ca^2+^ (carry out Ca^2+^/CaM signaling) ions are there (Vafadar et al., 2020a,b,c).

Under stress conditions, H_2_O_2_ and NO interact with Ca^2+^ ions forming an intricate signaling web to withstand salinity (Hasanuzzaman et al., 2018). Under salt stress conditions, it was observed that pre-treated quinoa seeds with an NO donor (sodium nitroprusside; SNP), a reactive oxygen species (H_2_O_2_), and CaCl_2_ showed a significant positive linear correlation with germination rate (GR) and germination index (GI), whereas a reversed linear correlation occurred between them with mean germination time (MGT) (Hajihashemi et al., 2020). The pretreatment enabled seed germination and rapid seedling establishment in the salt-affected soil. Pre-treating with NaCl completely prevented the decrease in the activities of alpha amylase and beta amylase. Salinity stress decreases seed germination by inhibiting the main enzymes, α- and β-amylase, which hydrolyze starch during germination, and pretreatment lowered this negative effect of salinity on these enzymes. Studies indicate that exposure to molecules, such as H_2_O_2_ or CaCl_2_, may reduce the adverse effect of environmental stress on amylase activity and restore normal germination (Zheng et al., 2009; Li et al., 2013; Li Z. et al., 2017; Bouallègue et al., 2017). The most significant finding from the study conducted by Hajihashemi et al. (2020) was that the presence of NO, H_2_O_2_, and Ca^2+^ resulted in enhanced amylase activity. The rise in starch degradation increases germination, and more seeds sprout (Li et al., 2013). This connection could alleviate the negative impact of salt stress on quinoa germination.

Polyamines (PAs), like putrescine, spermidine, and spermine, are well regarded besides their substantial plant developmental processes and adaptation toward environmental cues (Pathak et al., 2014). It has been established that PAs play a vital role in a variety of cellular pathways including programmed cell death. Expression levels of PA biosynthesis-related genes were shown to be modulated under stress (Gupta et al., 2013; Shi and Chan, 2014). The modulatory response of arginine decarboxylase (ADC) (regulatory enzyme of PA biosynthesis) to salinity is a key regulator of the adaptive response in plants (Liu et al., 2006). Transcriptomics of PA biosynthetic genes in different varieties of rice revealed that the ADC gene is induced under salinity stress (Do et al., 2014). Studies indicated that perhaps the PA metabolic pathway is in intricate crosstalk with other signaling pathways including ABA, H_2_O_2_, and gamma-aminobutyrate (GABA) (Marco et al., 2011; Seifikalhor et al., 2019). Within that direction, Kalhor et al. (2018) have demonstrated that GABA helps increase salinity tolerance in lettuce. PA metabolism further induces NO output, which has been linked to several other stress intermediaries like Ca^2+^ ions and protein kinases. Under salinity conditions, PAs can engage ion channel proteins thereby affecting their conductivity (Zhao F. et al., 2007; Velarde-Buendía et al., 2012). Garufi et al. (2007) proposed that PAs perform through regulating the activity of multiple ion channels indirectly by enhancing interactions with “14–3–3 proteins” (a family of highly conserved regulatory molecules). In stressful conditions, it is possible that high levels of cytoplasmic Ca^2+^ can be deleterious to standard cellular metabolism. Since active Ca^2+^ efflux networks play a pivotal role in sustaining cell Ca^2+^ contents, PAs activate Ca^2+^ efflux mechanisms, such as the PM channel and the membrane Ca^2+^-ATPase, while maintaining steady plasma Ca^2+^ levels (Pottosin et al., 2012, 2014; Pottosin and Shabala, 2014). In this regard, PAs metabolism is related to Ca^2+^ signaling indirectly, even though underlying regulatory mechanisms remain unclear.

Several studies indicate the control of NO metabolism during salt tolerance (Wimalasekera et al., 2011; Ahmad et al., 2016). As a major NO production pathway, NO synthase (NOS) enzyme leads to most of the NO production in animals (Santolini et al., 2017) and plants (Moreau et al., 2008). When realizing that NO is a molecule linked to PAs via the common precursor of 1-arginine, it could be probable that PAs like spermidine and spermine produce NO in plants. The role of NO in signaling may be influenced by mobilization of intracellular calcium or interaction with calcium channels which ultimately elicit Ca^2+^ signaling (Courtois et al., 2008). These underlying mechanisms of Ca^2+^ and NO signaling affect each other. NO synthesis mediated by NOS operates via Ca^2+^ and CaM signaling (Corpas et al., 2004, 2006). Lamotte et al. (2006) found that NO plays a massive part in the activation of plasma membrane channels and the subsequent release of Ca^2+^ under salt stress recovery.

### ROS Crosstalk

The ‘salt overly sensitive’ (SOS) pathway could potentially play an important role in the membrane conductance of the root epidermal cell of plants to the extracellular acidic environment, thereby helping to detoxify this ion in the root epidermal cells and thereby extruding this ion form the root epidermal cells (Quintero et al., 2002; Martínez-Atienza et al., 2007). The “salt overly sensitive” (SOS) pathway forms a critical pillar for preserving ion homeostasis when exposed to salinity stress (Ji et al., 2013). Nevertheless, during salinity, sustained accumulation and scavenging of ROS serve mostly as distress indicators, whereas redox homeostasis as well as antioxidant signaling at the cellular levels are engaged in stress sensing and tolerance. However, high levels of ROS accumulation can impair essential plant metabolic processes and development (Gill and Tuteja, 2010; Bose et al., 2014; Jajic et al., 2015). Electrons released by oxidants serve as a signaling cue in the cell to alert the plant to stress adaptation (Mittler et al., 2011). Salinity stress also causes ROS-mediated damage to lipids and proteins, and contributes to programmed cell death (Poór et al., 2012). Yet, ROS and calcium (Ca^2+^) are considered to be effective intracellular signals (Gilroy et al., 2014). In response to a high salinity environment, cytosolic calcium increases, which stimulates calcium sensors and calcium signaling pathways (Boudsocq and Sheen, 2009). However, plants also employ ion transport control (e.g., sodium and potassium accumulation), compatible solute aggregation, and expression of genes under salt stress (Kurusu et al., 2015).

Reactive oxygen species are continually formed in plant organelles as inevitable byproducts of metabolic activities (Apel and Hirt, 2004; Abogadallah, 2010). Although, the increased oxidative stress that would lead to cell death through apoptosis is preventable and can be counteracted with antioxidants, it would also be a point of differentiation. It is clearly right, as many plants often undergo necroptosis, including those that do not typically reside in a stressful climate (Coll et al., 2011). In comparison, singlet oxygen is utilized as a substrate of lipoxygenases activating a metabolic cascade that will produce a further essential stress signal, JA (Farmer and Mueller, 2013). Furthermore, ABA synthesis is also triggered by ROS (Xiong and Zhu, 2003). Plant species under salinity or drought stress will close their stomata and thus reduce moisture loss and their CO_2_ influx (Hsu and Kao, 2003). Subsequently, carbon reduction and photosynthetic NADPH utilization by the Calvin cycle decreases, resulting in the development of electron holes in photosystem I that leads to electron leakage to O_2_ (Türkan and Demiral, 2009).

Peroxisomal glycolate oxidase is a major source of ROS that is involved both in basal conditions and stress conditions (Mittler, 2002). ROS quenching can also be accomplished by other signaling molecules such as NO. NO nullifies Fenton-type oxidative stress by scrounging superoxide thus inhibiting the development of oxidizing agents that leads to recovery of redox homeostasis (Lamattina et al., 2003). As an alternate regulator of NO and GSH, H_2_S, recently identified as a signaling molecule in crops, enhances GSH concentrations, affects enzymatic activity, and communicates with NO (Paul and Snyder, 2012; Lisjak et al., 2013). The NO formed by the phytohormone will serve as a crosstalk intermediary between the two signaling pathways. This signal attenuation is comprehensive, since even ROS derived from NADPH oxidase activity in the plasma membrane are vital to trigger ABA signaling (Kwak et al., 2003).

Reactive oxygen species aggregation throughout stress conditions often depends on the capabilities of ROS recycling through the scavenging system. The buildup of different reactive oxygen forms gradually contributes to the adaptability to stress factors and culminates in apoptosis. SA administered through the root system resulted in a raised H_2_O_2_ concentration in young leaf and root tissue which caused plant death (Gémes et al., 2011). Szepesi et al. (2009) observed that even under salinity, plants pre-treated with SA contained even more Na^+^ than controls. The leaves did not exhibit any signs of salt damage, and rather, their photo- and membrane-integrity remained intact. As intracellular ROS is hugely involved in salt responses and active oxygen radical production within the cell is sometimes correlated with abiotic stress, it was of concern whether intracellular ROS and NO could make a significant contribution to the loss of vitality of distressed leaf protoplasts.

Reactive oxygen species-activated calcium-permeable channels including “respiratory burst oxidase homolog” (Rboh) are being hypothesized to entail an optimistic feedback loop that activates calcium ion and active radical signals in root cells (Takeda et al., 2008). RbohC/RHD2 has an affect on the production of ROS and controls Arabidopsis root hair elongation in a Ca^2+^-dependent fashion (Takeda et al., 2008; Monshausen et al., 2009). Salinity-induced (Ca^2+^) cyt is enhanced through hyperpolarization- and depolarization-activated PM Ca^2+^-permeable channels (Tracy et al., 2008). Salinity-induced escalation of cytosolic Ca^2+^ entry plays an important role in ROS signaling and saline tolerance (Shabala and Newman, 2000; Kader and Lindberg, 2010). Polyamines induced by salinity can produce oxygen-derived free radicals as substrates for apoplastic ROS enzymes (Kärkönen and Kuchitsu, 2015). Both OH and polyamines may significantly change the cationic and anionic conducting pathways and affect Ca^2+^ signaling in plants (Pottosin et al., 2014). It can unwrap a novel feature of ROS development during salinity adaptation and/or the acclimation process. A notable research paper demonstrates that perhaps the recruitment of Ca^2+^/CaM-like proteins (CDPKs) are far more pronounced in halophytes compared to glycophytes which experiences salt stress (Xu P. et al., 2013). Such calcium-binding proteins can play a pivotal role as critical amplifiers of initial calcium influx under salt stress. Ca^2+^ signaling is also very important in seawater stress’s impact on signaling mechanisms. It is suggested that TPC1 is engaged in the dissemination of salt stress cues and seems also to participate in the plant defense response (Choi et al., 2014). Inflammation triggered by Ca^2+^-ROS pulses concerning TPC1 can be one of the plant stresses tolerating mechanisms (Choi et al., 2014; Gilroy et al., 2014). Furthermore, NO has been demonstrated to have potent antioxidant activity; preventing and minimizing lipid peroxidation and protein oxidation (Fancy et al., 2017). Saline conditions enhance NO output that minimizes salinity-induced damages (Fatma et al., 2016; Da Silva et al., 2017). At about the same time, numerous sources claim a preventive action for NO in salt-stress tolerance due to upregulating antioxidant activity in various native plants (Zheng et al., 2009; Sheokand et al., 2010).

### Plant Growth Regulators Crosstalk

The productivity of agricultural crops has continuously and adversely been affected by soil salinity. A plant’s ability to tolerate salinity stress can be augmented by application of signaling molecules such as H_2_S, NO, and H_2_O_2_. For example, exogenous application of NO decreases the accumulation of Na^+^ and stabilizes the concentration of mineral nutrients, and thereby results in a balanced photosynthetic rate and re-establishment of vegetative growth in lettuce (*Lactuca sativa*). Osmotic regulation, activation of the antioxidant system, and balanced phytohormones help to increase plant tolerance against salinity stress (Campos et al., 2019). Salinity stress adversely affects plant growth through upregulation of proline, MDA, and ABA content while downregulating K^+^/Na^+^ ratio and electrolyte leakage. Exogenous application of sodium nitroprusside (SNP) and 24-epibrassinolide (EBL) in combined form increases the endogenous level of ABA in Indian mustard (*Brassica juncea* var, Varuna) through proline, nitrogen, and ABA metabolism (Gupta et al., 2017). Soil salinity has a detrimental effect on agricultural crops through water deficiency and modified K^+^/Na^+^. This process leads to altered cellular redox pathways by producing reactive oxygen species such as H_2_O_2_, superoxide (O_2_^–^), and hydroxyl radicles (^•^OH). These free radicals pose a severe toxic impact on a molecular, biochemical, physiological, and cellular level via the lipid peroxidation pathway and lead to protein and nucleic acid destruction, and thereby negatively affect several vital pathways such as gaseous exchange, plant growth and development, and proline and nitrogen metabolism (Siddiqui et al., 2012; Gupta et al., 2017). The crosstalk of PGRs with other signaling compounds under salinity stress tolerance are illustrated in Table 2. NO metabolism regulates several biochemical pathways of ABA homeostasis in plants such as seed germination, dormancy, leaf senescence, stomatal movement, and fruit ripening in normal and stress conditions. The signaling molecule NO induces post translational modifications such as tyrosine nitration and sulfur-nitrosylation of proteins which regulate ABA signaling pathways. NO modulates antioxidant systems such as SODs and the catalase and ascorbate GSH cycle, and also affect ABA-induced reactive oxygen species production (Prakash et al., 2019). Phytohormones play a critical role in plants to adapt them to an unfavorable environment such as salinity via modulating physiological responses. Both phytohormone-ethylene and GAs play crucial roles to mitigate salinity stress by activating defense regulatory genes or increasing plant growth. However, both ethylene and GA are interconnected with each other; GA is well known to increase ethylene synthesis while its signaling is also dependent on ethylene (Iqbal et al., 2012). Transcript-based meta-analysis studies have illustrated that both ethylene and GA metabolism-related genes are expressed in plants under salinity stress. The precursor ACC may be synthesized unanimously for ethylene and GA synthesis. Exogenous application of ethephon and GA_3_ reduces the adverse effect of salinity on seed germination of *Amaranthus caudatus* (Bialecka and Kepczynski, 2009). Ethephon showed a more stimulatory effect on seed germination than GA_3_ under soil salinity. Foo et al. (2006) studied the interaction effect of ethylene and GA synthesis in pea and revealed that ethylene synthesis was negatively controlled by phytohormones and also tended to reduce GA production. Similarly, ethylene and GA have a positive effect on hypocotyl elongation in Arabidopsis (De Grauwe et al., 2007). It has been reported that GA alone is ineffective but acts synergistically with ethylene and promotes the number of penetrating roots and growth rate of emerged roots (Steffens et al., 2006). Their effect is not additive in nature, but both are synergistic with each other.

**TABLE 2:** The crosstalk of plant growth regulators with crucial signaling compounds under salinity stress and their salt tolerance mechanism in different crops.

Crosstalk	Crop	Tolerance mechanisms	References
NO-Melatonin	*Brassica napus* L. zhongshuang 11	Promotes seedlings root growth, maintains redox balance, lowers the Na^+^/K^+^ ratio, and modulates the antioxidant defense genes, *NHX1*, and (*SOS2*) transcripts	Zhao et al. (2018)
NO-Salicylic acid (SA)	*P. sativum* L. (var. Shubhra IM-9101)	Improves seedlings radicle length, reduces oxidative stress by decreasing superoxide radicles and H_2_O_2_, induces the isoform transcript of SOD, POX, APX, and enhances osmolytes accumulation	Yadu et al. (2017)
NO-SA	*Capsicum annuum* L.	Regulates the enzymes of AsA-GSH cycle enzymes, lowers the Na^+^/K^+^ ratio and electrolyte leakage, reduces H_2_O_2_, MDA, and proline contents	Kaya et al. (2020)
SA-H_2_O_2_-Ca	vena nuda cv. North China No. 1	Improves shoot and root dry weight, improves SOD, CAT, GSH, and ascorbic acid, and reduces MDA contents	Xu et al. (2008)
24-Epibrassinolide-SNP	*Brassica juncea* L. cv. Varuna	Improves length and biomass of root and shoot, enhances leaf area, chlorophyll, and carotenoid contents, 51% decline in electrolyte leakage and 37% in lipid peroxidation, improves stomatal opening by enhancing length and width of stomatal aperture, maintains ion homeostasis, and lowers ABA content	Gupta et al. (2017)
SA-ROS-NO	*Solanum lycopersicum* cv. Rio Fuego	Reduces the production of ROS, improves cell viability, and readjusts polyamines	Gémes et al. (2011)
NO-Phytohormones	*Lactuca sativa*	Decreases Na+ accumulation, stabilizes mineral nutrient concentration, improves photosynthesis rate, activates the antioxidant system, adjusts osmotic and hormone balance	Campos et al. (2019)
ABA-JA	*Nicotiana tabacum* NC89	Improves photosynthetic efficiency, reduces photo damage, induces stomatal closure, and improves antioxidant defense genes	Yang et al. (2018)
Melatonin-Ca	*Dracocephalum kotschyi* Boiss.	Improves relative water, proline contents, and ion homeostasis, enhances antioxidant enzymes activities, and induce systematic salt tolerance via influencing other signaling compounds	Vafadar et al. (2020c)
Ethylene-H_2_O_2_	*Solanum lycopersicum* cv. Yuanbao	Improves seedling biomass, chlorophyll content, and photosynthetic rate, enhances brassinosteroids synthesis, and reduces oxidative damage by enhancing antioxidant enzymes	Zhu et al. (2016)

Seed germination is affected by both ethylene and NO under salinity in plants. Exogenous application of ACC (a precursor of ethylene biosynthesis) or SNP (an NO donor) inhibits the negative impact of salinity on seed germination of Arabidopsis (Lin et al., 2013). However, the stimulatory effect of both ACC and SNP was reduced by the inhibitor of ethylene biosynthesis, i.e., aminoisobutyric acid (AIB) or NO scavenger compound, i.e., 2-phenyl-4,4,5,5-tetramethyl-imidazoline-1-oxyl-3-oxide (cPTIO) and indicated the interaction effect of both NO and ethylene on seed germination under salinity. Moreover, NO production was increased by ACC and overexpression of the *ACS2* gene was noticed by SNP which is directly involved in ethylene biosynthesis. Thus, this indicates the importance of both in their production under salinity stress. Interestingly, exogenous application of ACC increased seed germination under oxidative stress induced by H_2_O_2_. However, NO-treated wild-type Arabidopsis plants were less affected and no effect was observed on ethylene-insensitive mutant seeds.

## Omics Technology in Salinity Stress Tolerance

**FIGURE 3:**
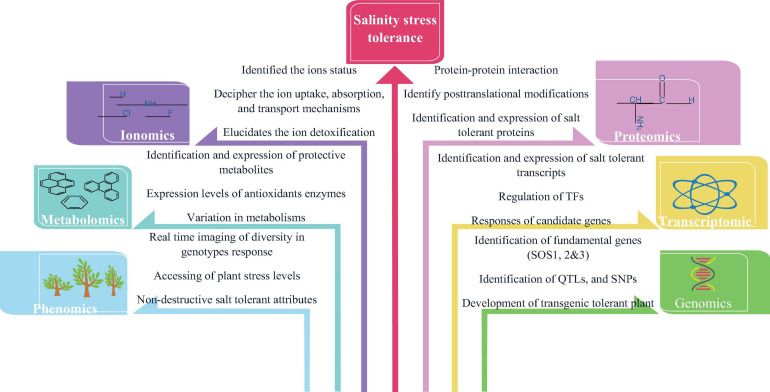
The application of different omics approaches in salinity stress tolerance.

The major influences on plant growth limitation in salt-affected soil are due to osmotic stress and ion toxicity (Munns and Tester, 2008; Bargaz et al., 2016). Plants adapt distinct strategies (molecular, biochemical, and physiological adjustments) to modulate metabolic pathways and at the same time, to combat cellular salt levels via regulating water and ion homeostasis. In this regard, the modern integrative “omics” approach in plant biology has taken momentum over the last two decades in the research area powered by advances in platforms for nucleic acid sequence, peptide sequencing platforms, mass spectrometry (MS), efficient computational skills, and data analysis methodologies. This integrated “omics” system offers a snapshot of cells, tissues or organisms’ developments, functions, and relationships by characterizing and quantifying all their biomolecules using a high-performance approach (Soda et al., 2015; Mosa et al., 2017; Parida et al., 2018). In recent years, omics technologies have shown promising results and been tested in numerous crops, which are highlighted in Table 3. Among them the application of some omics approaches under salinity stress tolerance are represented in Figure 3 and discussed below.

**TABLE 3:** The crucial genomics, transcriptomics, metabolomics, and proteomics approaches used in different crops.

Omics approach	Crop	Technology	References
Genomics	Arabidopsis	BAC (bacterial artificial chromosome) by BAC	Kaul et al. (2000)
	Rice	Whole-genome sequencing analysis Genome-wide meta-analysis	Subudhi et al. (2020), Mansuri et al. (2020)
	Barley	BAC by BAC Hierarchical shotgun sequencing Roche/454 pyrosequencing Genome-wide association study	Schulte et al. (2009), Wicker et al. (2009), Mwando et al. (2020)
	Maize	BAC by BAC	Pennisi (2008), Schnable et al. (2009)
	Poplar	Whole-genome shotgun sequencing (WGS)	Tuskan et al. (2006)
	Grape	WGS	Jaillon et al. (2007)
	Papaya	WGS	Ming et al. (2008)
	Sorghum	WGS	Paterson et al. (2009)
	Soybean	WGS	Schmutz et al. (2010)
	Apple	Genome-wide duplication (GWD)	Velasco et al. (2010).
	Potato	WGS	Potato Genome Sequencing Consortium (2011)
	Mango	PAC biosequencing	Singh et al. (2014)
	Peach	WGS	Ahmad et al. (2011)
	Banana	WGS	D’Hont et al., 2012
	Tomato	WGS	https://www.wur.nl/en/show/Sequencing-of-the-tomato-genome.htm
	Cucumber	WGS	Huang et al. (2009).
	Wild strawberries	Roche/454, Illumina/Solexa and Life Technologies/SOLiD platforms, next-generation sequencing (NGS)	Shulaev et al. (2011)
	Musk melon	NGS	Garcia-Mas et al. (2012)
	Watermelon	NGS	Garcia-Mas et al. (2012)
Transcriptomics	Rice	Micro array SAGE (serial analysis of gene expression)	Li et al. (2006), Matsumura et al. (1999)
	Wheat	DNA array	Jauregui et al. (2015)
	Maize	RNA sequencing	Zhao et al. (2019).
	Barley	RT-PCR (real time polymerase chain reaction)	Svensson et al. (2006)
	Arabidopsis	SAGE	Ekman et al. (2003)
	Cassava	ESTs (expression sequence tags)	Suarez et al. (2000)
	Peas	RNA display analysis	Lapopin et al. (1999)
Proteomics	Rice	2D-GE (2-dimension gel electrophoresis) Isobaric Tags for Relative and Absolute Quantitation (iTRAQ)	Zi et al. (2012), Xu et al. (2017), and Lakra et al. (2019)
	Maize	2D-GE iTRAQ	Amara et al. (2012), Luo et al. (2018), and Chen et al. (2019)
	Wheat	2D-PAGE (*Polyacrylamide gel electrophoresis)* MS/MS (mass spectrometry)	Amiour et al. (2002), Singh et al. (2017)
	Barley		Süle et al. (2004)
	Soyabean	Mass spectrometry	Galant et al. (2012)
	Tomato	2D-PAGE	Afroz et al. (2009)
	Sugar beet	Liquid chromatography-tandem mass spectrometry (LC-MS/MS)	Hajheidari et al. (2005)
	Grape berry	2D-PAGE	Di Carli et al. (2011)
	Peanut	LC-Q-TOF (MS/MS)	Chassaigne et al. (2007)
	Apple	2D electrophoresis and IgE-reactivity. *Electrophoresis*	Herndl et al. (2007)
	Pear		Pedreschi et al. (2008)
	Peach		Zhang L. et al. (2011)
	Populus	Shotgun MS/MS profiling	Kalluri et al. (2009)
	Stone fruit	2D PAGE	Abdi et al. (2002)
Metabolomics	Rice	GC-MS, NMR (nuclear magnetic resonance)	Gayen et al. (2019), Ma et al. (2018)
	Maize	HNMR, GC-MS	Gavaghan et al. (2011), Zörb et al. (2013)
	Wheat	GC-MS, HPLC, GC-TOF/MS	Guo R. et al. (2015), Borrelli et al. (2018), and Che-Othman et al. (2019)
	Barley	TIC (total ion chromatogram)	Shelden et al. (2016)
	Arabidopsis	RHPLC (high performance liquid chromatography)	Arrivault et al. (2009)
	Tobacco	NMR	Zhang J. et al. (2011)
	Tomato	UHPLC-ESI/QTOF-MS	Rouphael et al. (2018)
	Nitraria	GC-TOF/MS	Ni et al. (2015)
	Lotus	GC-TOF/MS (gas chromatography- time-of-flight mass spectrometry)	Sanchez et al. (2011)
Ionomics	Alfalfa Halophytes Lotus Barley	High-throughput sequencing, element-specific profiling, mass spectrometry, deletion mapping, X-ray fluorescence, neutron activation analysis, DNA microarray, and bulk segregant analysis	Baxter (2009), Becker and Becker (2010), Sanchez et al. (2011), Wu et al. (2013), Huang and Salt (2016), and Arshad et al. (2017)
Phenomics	Rice	PHENOPSIS WIWAM	Granier et al. (2006), Humplík et al. (2015), and Meng et al. (2017)
			

### Genomics

It is very crucial to understand the plant genomic response toward environmental stress. Plants are acutely complex and consist of a large number of genes in the reaction to salinity. It has been difficult to absolutely understand how plants react to salinity because of their multi-genetic nature. Genomics has made considerable strides over the past decade and has played a critical role in delivering the information needed to promote crop production. Genomics is a branch of “omics” that studies a certain genome and discloses useful knowledge on the organism’s biology (Gilliham et al., 2017). By genomics, the genes involved in salinity stress response have been identified and characterized; signaling pathways have been mapped and certainly this information can be used for salinity tolerance of existing plants. It is significant to mention that genomics as a tool primarily improves and does not replace current technologies. Several high-throughput approaches, such as forward genetics, candidate gene approach, serial analysis of gene expression (SAGE), expression sequence tag (EST), next generation sequencing (NGS), high resolution melting (HRM), targeting-induced local lesion in genomes (TILLING), RNA interference (RNAi), and genome wide association study (GWAS), etc. have enabled us to not only understand salinity tolerance in plants but have also opened the path for developing plants under salinity stress. Using forward genetics, fundamental genes (*SOS1*, *SOS2*, *SOS3*) of the salt tolerance pathway have been identified (Zhu et al., 1998) and this knowledge of candidate gene approach has been pursued to efficiently identify the SOS gene orthologs in rice (Martínez-Atienza et al., 2007), Poplar (Tang et al., 2010), and tomato (Olías et al., 2009). Eleven single nucleotide polymorphisms (SNPs) were identified in the coding region of five salt-tolerant rice genotypes by exploring the advance method of TILLING, i.e., Eco-TILLING (Negrão et al., 2013). Because of its ability to boost the resolution of quantitative trait loci (QTL) detection without spending additional efforts in population development, GWAS receives greater attention. Recently, three novel QTLs were identified on chromosomes 4, 6, and 7, which are associated with salt tolerance in rice, through the use of molecular breeding approach GWAS (Kumar et al., 2015). Salinity tolerance may also be correlated with six genomic regions in soybean confirmed through SoySNP50K BeadChip iSelect (Huang et al., 2018). Consequently, nine SNP-rich regions were identified in 215 accessions of Asian cotton using the same GWAS approach as those related to plant parameters in salt stress conditions (Dilnur et al., 2019).

The use of RNAi technology in salt tolerances shows the positive control of tocopherol cyclase (Ouyang et al., 2011). New technologies of genomics like zinc finger nuclease (ZFN), transcription activator-like effector nucleases (TALENs), CRISPR-Cas9 (Pennisi, 2013), and Speed Breeding (Li et al., 2018) provides the opportunity to generate precisely engineered crops for salinity tolerance. The latest study of genotyping-by-sequencing in cowpea has been used to discover the effects of salinity tolerance on seed germination and seedling growth (Ravelombola et al., 2018). Nine haplotypes, two salt-tolerant and seven salt-sensitive, were addressed by a new genome sequencing experiment of 31 landraces and 22 wild soybeans (Guan et al., 2014).

### Transcriptomics

Soil salinization is recognized as a major problem for agricultural production and sustainability at a global level. The mechanisms of salinity tolerance are well known to be complicated and governed by polygenic traits (Munns and Tester, 2008). Therefore, improvements in understanding other “omics” beyond genomics have helped assign functional roles to candidate gene(s)/QTL(s) that relate to multiple abiotic stresses including salinity stress in crop plants (Salt et al., 2008). Another branch of “omics” technology is transcriptomics which deals with the RNA expression profile of organisms at temporal and spatial bases (Duque et al., 2013; El-Metwally et al., 2014; Shen et al., 2019). Unlike genomics, the transcriptome is highly complex and dynamic, and changes depending on diverse factors (El-Metwally et al., 2014). Recent shifting of RNA sequencing (RNA-seq) high-performance technology from the microarray accelerated the response of the candidate gene to stress (Liu et al., 2014; Vu et al., 2015; Conesa et al., 2016). The recently developed transcriptomics measure the abundance of transcripts of thousands of genes in parallel. RNA profiling is currently being carried out by means of RNA sequencing, microarray platforms, digital gene expression profiling, and SAGE (Molina et al., 2011; Raney, 2012; Duque et al., 2013; Xu Y. et al., 2013; Leisner et al., 2017; Li P. et al., 2017; Kreszies et al., 2019). This technology improves the ability, in salt stress, to identify transcripts/genes that are essential in controlling transcription and translation machinery (Sahi et al., 2006; Jamil et al., 2011).

To understand the transcriptomic changes during salt stress, RNA sequencing (RNA-Seq) has become the most used method for identification of novel genes and their expression pathways (Hrdlickova et al., 2017). Transcriptome profiling has been extensively and successfully used to analyze salt stress response mechanisms of plants. It is an effective method to find common sets of genes that are differentially expressed between stress-tolerant and sensitive genotypes with diverse genetic backgrounds (Peng et al., 2014). Comparing the difference in transcriptional levels between tolerant and sensitive genotypes under stress conditions, the genes related to stress tolerance can be isolated. A transcriptomics study also revealed different up- and downregulated transcriptional factors such as MYB, MYB-related, AP2-EREBP, NAC, and WRKY (Chen F. et al., 2020). This transcriptional profiling study gives a better insight into the understanding of the key components in the plant salt tolerance network which is important for developing more salt-tolerant plants. NGS and SAGE techniques were employed together by Molina et al. (2008, 2011) to classify the entire chickpea salt transcriptome. Likewise, for *Arabidopsis thaliana* responses to salt stress, Rasmussen et al. (2013) used large-scale microarray analysis. The comprehensive genome-wide study of common beans was used to recover a total of 155 bHLH (helix loop helix) genes related to salt stress response (Kavas et al., 2016). A research was performed with Solexa/Illumina to investigate the transcriptome expression profiles for Poplar (*Populus simonii* X *Populus nigra*) under salinity stress (Chen et al., 2012). Similarly, differential expression levels were checked in salinity stress for WRKY-TF genes (Garg and Singla, 2016). Most currently, miR156 working in reaction to salinity stress in alfalfa was shown by Arshad et al. (2017).

### Proteomics

Salt stress is predicted to cause salinization of arable lands within the next 25 years, which may result in 30% land loss and up to 50% by the year 2050. Salinity stress causes different genes to be expressed and the result is reflected in the protein profiles. It could thus be essential to collect whole proteins created by various abiotic stresses, including salt stress, to enhance our knowledge of protein networks associated with salt-interacting pathways (Ji et al., 2016). Recently developed “omics” technologies are being designed in plant sciences to determine key proteins or metabolites that are novel, covering metabolomics, proteomics, and genomics responsible for plants stress tolerance and also biomolecules that regulate the genes. These omics studies give us a better insight into the agents affecting plant growth and development. Proteomics deals unshakably with the identification of proteins, expression profile, post-translational modifications (PTMs), and protein–protein interactions underneath stress and non-stress conditions. Proteomics studies offer a new replacement approach to obtain proteins and pathways related to crop physiological and stress responses. Thus, determining plants at proteomic levels might facilitate the finding of pathways concerned in stress tolerance.

Plant responses to salt stress through the proteomics approach have been studied in both glycophytes and halophytes. Plant scientists have worked with model plants under saline stress at proteomic levels, Razavizadeh et al. (2009) in *Nicotiana tabacum*, Chen et al. (2012) in *Populus cathayana*, Chattopadhyay et al. (2011) in grass pea, and Xu C. et al. (2011) in *Agrostis stolonifera*. Moreover, agricultural plants have also been examined under saline stress in different analyses, e.g., durum wheat (Peng et al., 2009; Jacoby et al., 2010), canola (Bandehagh et al., 2011), sugarbeet (Wakeel et al., 2011), soybean (Sobhanian et al., 2010), peanut (Jain et al., 2006), S. bicolor (Swami et al., 2011; Ngara et al., 2012), maize (Zörb et al., 2010), tomato (Chen et al., 2009; Manaa et al., 2011), potato (Aghaei et al., 2008), and cucumber (Du et al., 2010), etc.

Plant roots exhibit the foremost negative symptoms of salt stress because the few genes that are responsive to salinity stress are induced more in roots than in shoots as evident from the findings of different workers in soybean, rice, wheat, maize, and potato (Hasanuzzaman et al., 2013). A proteomics study in soybean was carried out under salt stress with the use of different tissues. They recognized that 50S ribosome protein was downregulated in leaves and that it was thought to participate in the biosynthesis of soybean protein and cause a decrease in plant growth. A phosphoproteome study of the roots of rice on exposure to NaCl (150 mM) for a few hours by using Pro-Q Diamond stain revealed that 20 proteins were upregulated and 18 downregulated. They positively identified 17 of the 20 upregulated proteins and 11 of the 18 downregulated ones. Proteins related to GST, Hsp70, and mannose binding rice lectin were upregulated, while protein kinase, ATP synthase beta-chain, GALP hydrogenase were downregulated. They believed that phosphorylated proteins could be identified using Pro-Q Diamond stain under saline conditions. Of all proteins, 17 overexpressed proteins were responsive to salinity, however, some other proteins identified did not express in any of the proteomic reports on rice on exposure to salinity (Chitteti and Peng, 2007).

### Metabolomics

Higher plants have an excellent capacity to synthesize a broad variety of different molecules and play an important role in chemical defenses against biotic and abiotic stress. The synthesis and accumulation of all small molecule’s metabolites (less than 1.5 kDa) is an evolved, conserved, and ubiquitous process that shows immense variety in chemical structure and function known as metabolome, analogous to transcriptome, and proteome. Metabolism is an effective plant physiology method that is closer to phenotype than genes and proteins in response to abiotic stresses and metabolites, quite accurately representing the overall effects of genetic expressions and complex regulatory procedures (Scherling et al., 2010; Arbona et al., 2013; Ramalingam et al., 2015). Metabolites include a number of organic acids, hormones, amino acids, ketones, vitamins, and steroids. In this regard, metabolomics (i.e., the study of the metabolome, or the set of metabolites found in a given plant tissue or organ) play an essential role in procuring metabolic fingerprints or metabolic profiles based on the physiochemical properties of each metabolite using different test instruments and separation technologies (Jogaiah et al., 2013).

Relative to proteome and transcriptomics, this method generates more reliable information (Dos Santos et al., 2017). The illustration of stress tolerance mechanisms and metabolite profiling in plants has been improved by progress in mass spectrometry liquid chromatography or gas chromatography (LC-MS and GC-MS), high performance liquid chromatography nuclear magnetic resonance (NMR), direct injection mass spectrometry (DIMS), and other metabolomics techniques (Wolfender et al., 2013; Parida et al., 2018). Different researchers have noticed that variations in metabolites involving carbohydrate metabolism, tri carboxylic and glycolytic acid, amino acid biosynthesis, and at other protective antioxidant levels under stress are easily analyzed using metabolomics (Kumari et al., 2015; Jiao et al., 2018). A study of Lu et al. (2013a) revealed that a comparative study between soybean wild-type W05 and cultivated soybean C08 indicated abundance at a metabolic level of several compounds in a wild form, such as disaccharides, sugar alcohols, and acetylated amino acids. The increase of tocopherol in maize shoots and the sharp decrease in ascorbic acid levels after salt stress were reported by AbdElgawad et al. (2016). Wang et al. (2015) also stated that in *Kosteletzkya virginica* seedlings, proline levels increased when introduced to high salinity. In a study by Shen al. (2016), a decrease in the levels of glycolysis pathway-associated sugars occurred in barley in response to salt stress. A review of metabolomics comparison reported by Jiao et al. (2018) in common wild-type soybean W1 and W2 salinity-tolerant wild-type soybean revealed increased accumulation of various organic acids, TCA cycle metabolites, and various amino acids, which in turn gave W2 greater tolerance to salinity than W1. The study of metabolome data from foxtail millet roots showed in this research that 17 associated genes of flavonoid biosynthesis were significantly raised 2- to 11-fold under salinity in Yugu 2 (Pan et al., 2020). Salinity stress-specific metabolites could therefore serve as biomarkers to evaluate a salt-tolerant and sensitive genotype.

### Ionomics

The “ionome” is said to be the mineral and elemental collection of an organism (Salt et al., 2008). A landmark in ionomics research was identified in *A. thaliana* (Hirschi, 2003; Rea, 2003) where more than a thousand plants were analyzed for ionomic mutants. Ionomic circuits in crops are orchestrated and require critical reviews for high efficiency elemental profiling (Salt et al., 2008; Baxter, 2009). High-throughput sequencing, element-specific profiling, mass spectrometry, deletion mapping, X-ray fluorescence, neutron activation analysis, DNA microarray, bulk segregant analysis, and various reverse genetic tools confirmed the involvement of multiple regulators that regulate the ionome (Baxter, 2009; Becker and Becker, 2010; Sanchez et al., 2011; Wu et al., 2013; Huang and Salt, 2016). This has provided another possible avenue for exploration for plant-based genetic engineering for stress tolerance. Ionomics could lead to better management of root mineral nutrients status in plants (Shelden and Roessner, 2013). Ionomics has been deciphering the key elucidation toward ion homeostasis and ion detoxification in response to salinity stress in crops (Sanchez et al., 2011; Wu et al., 2013). The research findings indicate altered expression of calcium, magnesium, manganese, iron, and zinc within plants manifested with salinity stress. Phytogeographically plants reacted differently with increasing salinity. Research showed that a higher expression of salinity-responsive miR156 in alfalfa plants results in the accumulation of lower levels of Na^+^ (Arshad et al., 2017).

To adjust high salinity, plants both manage an ion uptake and distribution system (Sanchez et al., 2008). Since normal plant cells require high amounts of K1 and Na1, this ratio should be high. Low retention of K1 results in high K1 levels in the cytosol at higher Na1 concentrations that get depolarized through membrane currents and causes K1 efflux through outward-rectifying potassium (KOR) channels (Shabala and Mackay, 2011; Bose et al., 2014). Entry of Na1 into the cytosol occurs either via selective transporters or via cation channels, in a saltier environment (Sanchez et al., 2011). During salt stress, Ca^2+^ alters Na^+^ influx through extracellular channels and then Na1, K1, and Ca1 remain in balance through SOS pathways (Mahajan and Tuteja, 2005). Membrane transporters maintain appropriate levels of ions such as sodium (Na), potassium (K), hydrogen (H), and others (Shi et al., 2002; Maathuis, 2006; Sanadhya et al., 2015). To expel Na1 from the cell, SOS1 controls SOS3 along with a Ca21 sensor pathway (Khan, 2011). Through Ca21 messages, SOS3 experiences the extracellular salt environment. SOS2 activates the SOS system.

Na1 transport proteins keep Na1 concentrations low in cell cytosol. In soil, HKT1 contributes to the import of anions into plant roots, and the uptake of cations. The electrochemical potential results in accumulation of Na^+^ ions in the leaves in hyper-salinity environments (Su et al., 2003). The H1-ATPase complex consists of 11 heteromeric subunits (Shabala et al., 2014). By moving protons across the tonoplast of the endoplasmic reticulum (ER), V-type H1-ATPase produces the proton motive force which allows invisible influx of Na^+^ into the vesicle and thus lowers Na^+^ toxicity in the cell’s cytoplasm. Therefore, Na^+^ accumulation inside the vesicle of the cell emerged as an effective framework for osmotic pressure regulation in plant cells (Du et al., 2010). Halophytes have developed salt glands that remove surplus salts from metabolically active tissues (Agarie et al., 2007; Flowers and Colmer, 2008; Shabala et al., 2014).

### Phenomics

With the increasing output of genomics data, phenotyping ability does not offer a crucial benefit in regard to the understanding of phenotypically affected genetic variants which are significantly affected by the environment (Furbank and Tester, 2011). Multi-laboratory automated phenotyping systems are in development. Of the next-generation techniques, phenotyping techniques provided multiple advantages over traditional tests including non-destructive testing, automating data, and spectral imaging (Berger et al., 2012; Campbell et al., 2015; Al-Tamimi et al., 2016; Negrão et al., 2017). The technique for capturing molecular phenotypes at “high levels of detail, at unprecedented times and spatial scales” is gaining attention (Negrão et al., 2017). Some findings have been reported on the color of leaves, as well as stomatal responses to a variety of stresses applied to growing progeny (Berger et al., 2012; Hairmansis et al., 2014; Campbell et al., 2015; Awlia et al., 2016). Relative growth rate dynamics of chickpea plants under high salinity stress are notable and worth working on (Atieno et al., 2017). High-throughput phenotyping would be useful in assessing the impact of salt stress on photosynthesis, transpiration, ionic relationships, plant senescence, and on yield. Automatic and digital imaging of plants can further increase understanding of diversity of response of genotype to salinity. Besides, active vision cell technology, deep learning, and other modern phenotyping techniques may be used for defining, quantifying, and predicting plant salinity response with enhanced precision.

Salinity stress can affect seedling growth and seed germination. During vegetative growth, it decreases germination percentage, leaf area, total chlorophyll content, total biomass, and root and shoot length. Visible imaging is used for research, while RGB is used to classify the chlorophyll content of various samples (Mishra et al., 2016a, b). Next-generation phenotyping assays are used to determine salt tolerance, for example, “PHENOPSIS” (Granier et al., 2006) and “WIWAM”^1^ in rice and other field crops (Humplík et al., 2015; Meng et al., 2017).

## Conclusion and Future Perspectives

The regulation of plant growth and development processes under salinity stress is very complex. Its effect varies with the type of crop species, their growth habit, growth stages, and with environmental conditions. It affects germination to vegetative stage and up to maturity stage from very low to very high levels depending upon the mechanisms adapted or acclimatized by plants. At present, research on salinity stress tolerance is mainly based on the physio-morphological, biochemical, and molecular levels. The most promising ways to enhance salinity stress tolerance will be (1) screening of stress-tolerant genotypes, (2) a deep understanding of the effects and mechanisms of salinity stress in plants, and (3) identifying new genes, proteins, alleles, and transcription factors in respect to higher salinity tolerance. This study aims to understand the signaling mechanisms under salinity stress and we focused on the important signaling components MAPK and Ca under stress conditions. Thereafter, we addressed the crosstalk of important signaling compounds and plant growth regulators and cleared some ideas regarding their function and regulations under salinity stress. Then we discussed the role of recent advanced technology “omics” (genomics, proteomics, transcriptomics, and metabolomics) and how these technologies have helped in recent developments in salinity stress tolerance.

To address the challenges identified by existing research and studies, we came to following conclusions, which can be considered for future research in salinity stress tolerance.

(1) Plant stress tolerance is very complex, therefore existing strategies such as physiological, biochemical, soil, agronomical, and molecular approaches should be integrated to achieve salinity stress tolerance.

(2) Gasotransmitters and plant growth regulators have a crucial role in cell signaling, which needs to be focused on more to help understand the complexity of signaling pathways.

(3) “Omics” technologies are very promising to develop smart crops under environmental fluctuations. Integration of omics technology is a good choice for stress crop improvement programs.

Therefore, this study comprised recent progress in signaling mechanisms, crosstalk mechanisms of signaling compounds, and omics technology for salinity stress tolerance. These developments give a novel insight into understanding the signaling mechanisms and crosstalk under salinity stress tolerance and development of salinity stress tolerance genotypes by applying omics approaches.

## Author Contributions

All authors have prepared the draft of the manuscript. And also contributed during writing the manuscript, advised scientific suggestion as well as revised/edited the manuscript. All authors contributed to the article and approved the submitted version.

## Conflict of Interest

The authors declare that the research was conducted in the absence of any commercial or financial relationships that could be construed as a potential conflict of interest.

## Publisher’s Note

All claims expressed in this article are solely those of the authors and do not necessarily represent those of their affiliated organizations, or those of the publisher, the editors and the reviewers. Any product that may be evaluated in this article, or claim that may be made by its manufacturer, is not guaranteed or endorsed by the publisher.

## Footnotes

1
https://www.wiwam.be/
